# The Pulsed Electric Field Assisted-Extraction Enhanced the Yield and the Physicochemical Properties of Soluble Dietary Fiber From Orange Peel

**DOI:** 10.3389/fnut.2022.925642

**Published:** 2022-07-22

**Authors:** Rui Fan, Lei Wang, Jingfang Fan, Wanqiu Sun, Hui Dong

**Affiliations:** ^1^Department of Nutrition and Food Hygiene, School of Public Health, Peking University, Beijing, China; ^2^Key Laboratory of Agricultural Product Quality Evaluation and Nutrition Health, Ministry of Agriculture and Rural Affairs, Tangshan, China; ^3^Tangshan Food and Drug Comprehensive Testing Center, Tangshan, China; ^4^Hebei Plant Protection and Quarantine General Station, Shijiazhuang, China; ^5^Beijing Institute of Nutritional Resources Co., Ltd., Beijing, China; ^6^Shijiazhuang Institute of Pomology, Heibei Academy of Agriculture and Forestry Science, National Pear Improvement Centre, Shijiazhuang, China

**Keywords:** pulsed electric field, extraction, physicochemical properties, soluble dietary fiber, orange peel

## Abstract

The study aimed to investigate the effects of pulsed electric field (PEF)-assisted extraction on the yield, physicochemical properties, and structure of soluble dietary fiber (SDF) from orange peel. The results showed that the optinal parameters of PEF assisted extraction SDF was temperature of 45^o^C with the electric field intensity of 6.0 kV/cm, pulses number of 30, and time of 20min and SDF treated with PEF showed the higher water solubility, water-holding and oil-holding capacity, swelling capacity, emulsifying activity, emulsion stability, foam stability and higher binding capacity for Pb^2+^, As^3+^, Cu^2+^, and higher which resulted from the higher viscosity due to PEF treatment. Compared with the untreated orange peel, the SDF obtained with PEF exhibited stronger antioxidant activities, which was due to its smaller molecular weight (189 vs. 512 kDa). In addition, scanning electron micrograph images demonstrated that the surface of PEF-SDF was rough and collapsed. Overall, it was suggested that PEF treatment could improve the physicochemical properties of SDF from the orange peel and would be the potential extraction technology with high efficiency.

## Introduction

Orange peel, an easily available and inexpensive byproduct of the orange juice industry, is rich in dietary fiber (DF). The food industry produces ~100 million tons of orange peel each year in China (1). Unfortunately, most of it is not fully utilized and is discarded as industry waste causing environmental problems (2). Therefore, it is imperative to seek a potential technology to improve the added value of the by-products.

Dietary fiber, commonly used as a functional ingredient, possesses a variety of biological activities, including antioxidation (3), anticancer (4), and anti-inflammation (5). In particular, DF possesses strong water binding and alteration of viscosity, which in turn contribute to physiological attenuations, such as cholesterol and fat binding, decrease in blood glucose lipids and cholesterol levels, preventing constipation and facilitating good colonic health (6, 7). Besides, DF showed excellent physicochemical properties, including a gel-like property that contributes to food production and final products (8). Therefore, DF plays an important role due to its nutritional value and physicochemical properties (9). DF includes soluble dietary fiber (SDF) and insoluble dietary fiber (IDF). It is recommended to consume 25–35 g/d of DF, with a proportion of about 30–50% SDF (10); however, the average worldwide ingestion of DF is still considerably lower than the recommended daily intake levels. So, it is necessary to add DF to food products to increase the intake of DF. Orange peel, which contains more SDF than other sources, including cereals, is a good selection for producing SDF (2).

Pulsed electric field (PEF) technology applies short electrical pulses with high voltage to a product. When a living cell is moved into an electrical field, an electrostatic charge induces an electrical field potential. When the electrical potential reaches a critical value, reversible or irreversible electroporation is formed in the weak areas of the cell membrane, leading to the release of the cytoplasmic fluid and cellular materials (11, 12). Recently, many types of research have shown that PEF treatment can improve the extraction efficiency of bioactive compounds from plant tissues (13–15) and change their structure and physicochemical properties (16). Ma and Wang found the degree of esterification (DE) of pectin derivatives increased significantly with the PEF intensity from 18 to 30 kV/cm (17). Apoptosis tests proved that Morchella esculenta polysaccharide (MEP) extracted by the pulsed electric field (PEF) could inhibit the proliferation and growth of human colon cancer HT-29 cells in a time- and dose-dependent manner within 48 h (18). To better manufacture DF from orange peel, we need to understand how the DF may change under PEF.

Therefore, the aim of this study was to investigate the effect of PEF-assisted extraction on the yield of SDF and to determine the optimal parameters for obtaining SDF with PEF based on the physicochemical properties and structure. The results will provide the theoretical basis and technical support for the high-value application of the byproducts from the orange-producing industry in the future.

## Materials and Methods

### Materials and Reagents

The oranges [*C. sinensis* (L.) Osbeck] were bought from the supermarket in Tangshan. Moisture content was determined by drying samples at 100 ± 0.5°C to a constant weight, and the moisture measured was 5.00%. The dry orange peel sample was obtained by oven controlled at a constant temperature of 60°C for 4 h and then smashed to pass through a 10-mesh screen.

Heat-stable α-amylase (30 U/mg) and neutral protease (200 U/mg) solutions were purchased from Solebo Biotech Ltd. (Beijing, China). Standard monosaccharides, ferric chloride, acetate buffer, tripyridine triazine (TPTZ), ferrozine, 2,2′-azino-bis (3-ethylbenzothiazoline-6-sulfonic acid) (ABTS), and 1,1-diphenyl-2-picrylhydrazyl (DPPH) were purchased from Sigma-Aldrich (St. Louis, USA). All chemicals and reagents used were of analytical grade and purchased from Jiangtian Chemical Technology Co. Ltd. (Tianjin, China).

### Pulsed Electric Field Treatment for Fresh Orange Peel

**Figure 1:**
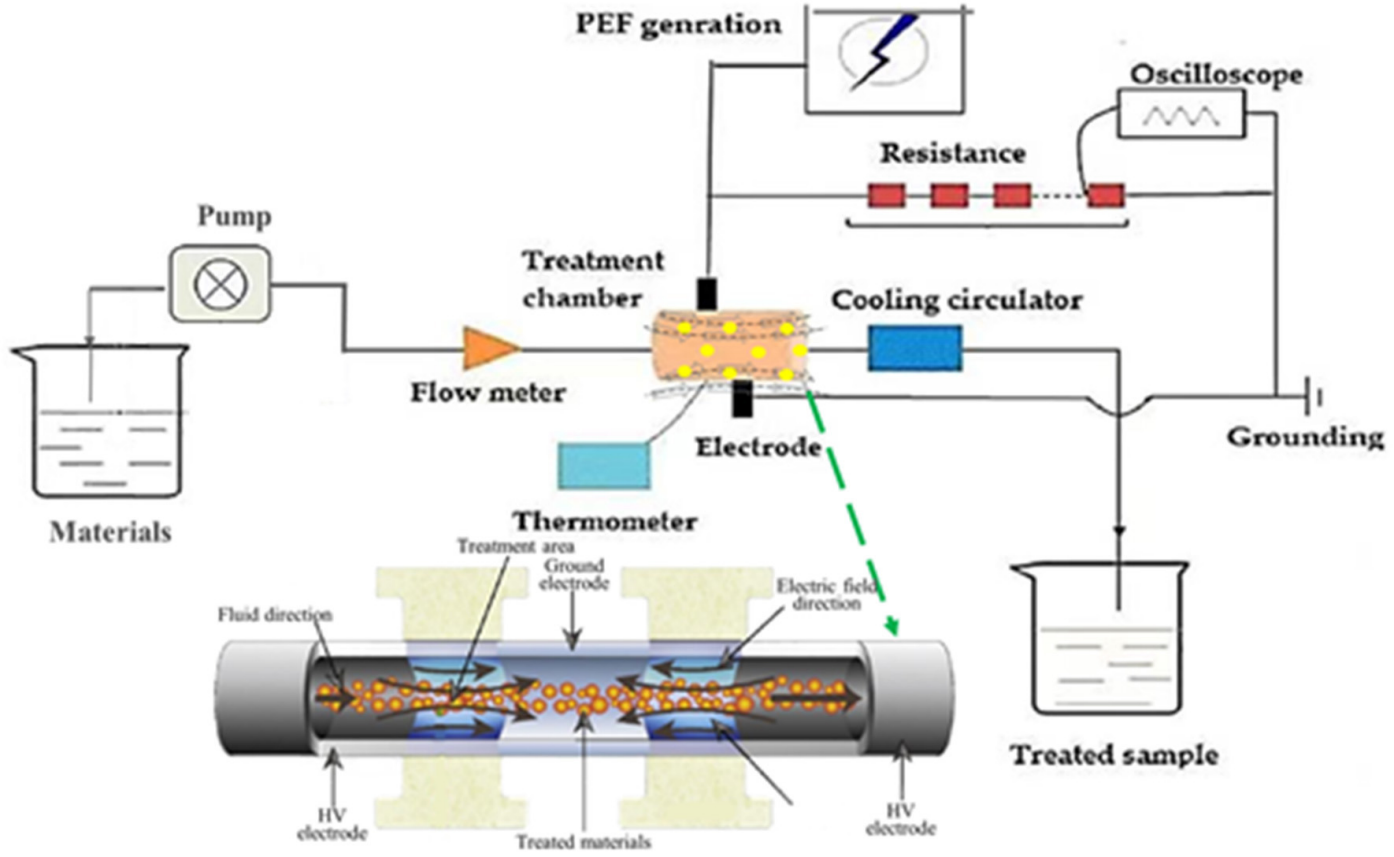
Schematic diagram of PEF system.

Pulsed electric field treatment was conducted using the EX-1900 PEF equipment (Xinan technology company, Guangzhou, China) with a batch 100 ml chamber (Figure 1). According to Kim's method (19), 5 g of fresh orange peel was put in 100 ml of deionized water at room temperature. PEF parameters were set as electrical field intensity from 2 to 10 kV/cm at the fixed number of 200, frequency of 1 Hz, and pulse width of 20 μs.

### Estimation of Cellular Damage

#### Determination of Cell Disintegration

The conductivity of water that immersed the fresh orange peel was determined in the estimation of membrane disruption induced by PEF.

The degree of fractured tissue was evaluated by electrical conductivity disintegration index *Z* value (20), which was calculated by Equation (1):


(1) $$\begin{array}{l} Z\text{=}\frac{\sigma \text{-}\sigma_{\text{i}}}{\sigma_{\text{d}}-\sigma_{i}} \end{array}$$

where σ is the electrical conductivity measured at each time point, σ_d_ is the electrical conductivity of completely disintegrated cells obtained by thawing after 24 h freezing, and σ_i_ is the electrical conductivity of untreated tissue.

#### Textural Profile Analysis

The textural profile analysis of the orange peel was estimated using TA-XT Plus Texture Analyzer (Stable Micro Systems, Godalming, UK). Following a previous report (21), the peel was subjected to compression with a trigger force of 0.04 N under a cylindrical probe (P/6) to a 5 mm-compressing distance at a 2.0 mm/s pretest speed, 1.0 mm/s test speed, and 2.0 mm/s *post-test* speed, and kept a rest period of 5 s between two cycles. All the measurements were undertaken at 25°C.

### The Preparation of SDF From Orange Peel

#### PEF-Assisted Extraction of the SDF

The PEF-assisted extraction of the SDF was performed based on the method from Boussetta, with some minor modifications (22). In total, 5 g of the dry orange peel powder and 75 ml of deionized water were mixed, and then, they were transferred to the PEF chamber. The orange peel was treated with different parameters, including electric field intensity (2.0, 4.0, 6.0, 8.0, and 10.0 kV/cm), pulse number (10, 20, 30, 40, and 50), liquid-solid ratio (5:1, 10:1, 15:1, 20:1, 25:1, and 30:1), and the temperature (25, 35, 45, 55, 65, and 75°C); meanwhile, the sample without PEF, i.e., 0.0 kV/cm and 0 pulses was used as the control. After the treatment, the mixture was filtered to obtain the SDF extract; then, it was separated by ethanol precipitation to detect the content of SDF.

#### Separation of SDF From Orange Peel

To determine the SDF content, the SDF of the extract was separated by an enzymatic gravimetric procedure based on the AOAC method 985.29 with minor modifications (23). The pH of the SDF extract was adjusted to 6.0, and 0.1 % (w/w) heat-stable α-amylase was added to hydrolyze at 95°C with 120-rpm stirring for 35 min. When the hydrolysate was cooled to 60°C, 0.016% (w/w) neutral protease was added to the hydrolysis for 30 min. Finally, the mixture was heated at 95°C for 5 min to finish the enzymatic reaction. The enzymatic hydrolysate was condensed to mix with 95 % (v/v) ethanol at 4°C for 12 h and subjected to centrifugation for 15 min at 4,200 rpm. The collected precipitate was dried and milled, which was the purified SDF.

#### Determination of Soluble Dietary Fiber

The soluble dietary fiber content was determined based on the AOAC (24). All measurements were performed in triplicate.

### Mathematical Modeling for the Kinetic of PEF-Assisted Extraction

In order to determine how to obtain the high-efficiency SDF under PEF with the least energy and time, Peleg's model, the most commonly used empirical model, was adopted to describe mathematically the variation in the SDF yield with time under different parameters (Equation 2) (25–28),


(2) $$\begin{array}{l} C\left(t\right)=C_{0}+\frac{t}{K_{1}+K_{2}\cdot t} \end{array}$$

where *C*(t) represents SDF yield at time *t* (mg/g), t represents the extraction time (min), *C*_0_ representing the initial concentration of SDF at time *t* = 0 (mg/g) was 0, and *K*_1_ and *K*_2_ represent Peleg's rate constant (min·g/mg) and capacity constant (g/mg), respectively. In fact, Equation (2) was simplified as follows:


(3) $$\begin{array}{l} C\left(t\right)=\frac{t}{K_{1}+K_{2}\cdot t} \end{array}$$

After deformation of Equation (3), we could plot the straight line between 1/Ct vs. 1/t based on Equation (4),


(4) $$\begin{array}{l} \frac{1}{C\left(t\right)}=K_{2}+\frac{K_{1}}{t} \end{array}$$

where *K*_1_, Peleg's rate constant (t), is calculated by extraction rate (*B*_0_) at the very beginning (*t* = *t*_0_) from Equation (5), and *K*_2_, Peleg's capacity constant, is calculated by the maximum of extraction yield (*C*_e_) at *t* reaching ∞ from Equation (6),


(5) $$\begin{array}{l} B_{0}=\frac{1}{K_{1}}\left(mg/g\cdot \text{min}\right) \end{array}$$

(6) $$\begin{array}{l} C_{t\to \infty }=C_{e}=\frac{1}{K_{2}}\left(mg/g\right) \end{array}$$

After *K*_1_ and *K*_2_are confirmed, _C(t)_ could be calculated based on Equation (4).

### Analytical Methods

#### The Processing Properties of SDFs

##### The Rheological Properties of SDFs

The rheological properties of SDF were measured with an AR-1500ex (TA Instruments, Delaware, USA). According to the previous report (29), the SDF solution (2 ml) was loaded onto the rheometer plate for equilibration before measuring. First, the linear viscoelastic region of all samples was measured by a stress sweep test (1 rad/s at 25°C); then, the frequency was swept inside the linear viscoelastic region from 0.01 to 10 rad/s at 1 Pa and 25°C to determine the frequency dependence of the storage modulus (G') and loss modulus (G”). In addition, the rheological parameters (shear stress, shear rate, and apparent viscosity) were obtained from the steady-state flow measurements performed from 0.01 to 300 s^−1^ sheet rate.

##### Solubility, Water-and Oil-Holding Capacities, and Swelling Capacity

The water solubility (WS) was determined based on the method reported by Zhang with minor modifications (30). A dry sample (1.0 g) was mixed with distilled water (50 ml) and stirred at 90°C for 30 min in a water bath, and then, it was centrifuged for 10 min at 4,200 rpm. The collected supernatant was freeze-dried and weighted. The WS was calculated using Equation (7):


(7) $$\begin{array}{l} WS\left(\%\right)=\frac{W_{1}}{W}\times 100 \end{array}$$

where W_1_ is the weight of supernatant after drying and W represents the weight of the SDF sample, respectively.

The water-holding capacity (WHC) of the SDF was measured based on Wang's method (31). Briefly, the SDF (0.5 g) was mixed with 5.0 ml distilled water and suspended for 30 s. After being kept for 24 h at 25°C, the mixture was centrifugated at 4,200 rpm for 15 min, and then, the sediment was collected and weighed to calculate the WHC from Equation (8):


(8) $$\begin{array}{l} WHC\left(g/g\right)=\frac{W_{1}-W_{0}}{W} \end{array}$$

where W_1_ is the total weight (g) of the centrifuge tube including SDF and water before centrifugation, W_0_ is the total weight (g) of the centrifuge tube including sediment, and W is the weight of the SDF sample.

The oil-holding capacity (OHC) of the SDF was measured using the method reported by Zhang (30) with slight modifications. Briefly, the SDF (0.5 g) was mixed with 5.0 ml olive oil, suspended for 30 s, and kept at 25°C for 6 h. Then, the mixture was centrifuged at 7,000 rpm for 15 min, removing the supernatant before the sediment was weighed to calculate the OHC from Equation (9),


(9) $$\begin{array}{l} OHC\left(g/g\right)=\frac{W_{1}-W_{0}}{W} \end{array}$$

where W_1_ is the total weight (g) of the centrifuge tube including SDF and water before centrifugation, W_0_ is the total weight (g) of the centrifuge tube including sediment, and W is the weight of the SDF sample.

The swelling capacity (SC) was determined based on the method reported by Zhang (32). The dry SDF (0.2 g) and 5 ml of water were added to a tube and hydrated for 18 h at 4°C. The final volume occupied by the SDF was recorded to calculate the SC using Equation (10):


(10) $$\begin{array}{l} SC\left(mL/g\right)=\frac{V}{W} \end{array}$$

where V represents the final volume containing the SDF sample and W represents the SDF sample.

##### Emulsifying Activity, Emulsion Stability, and Least Gelation Concentration

The methods to estimate the emulsifying activity (EA) and emulsion stability (ES) followed Chao with minor modifications (33). In total, 2 g of SDF was dispersed in 100 ml of deionized water and homogenized at 2,000 rpm for 2 min. Then, the corn oil (100 ml) was added and homogenized for 1 min to obtain the emulsion, which was centrifuged at 1,200 rpm for 5 min, and then, the emulsion volume was measured. The EA was calculated using Equation (11),


(11) $$\begin{array}{l} EA\left(mL/100mL\right)=\frac{V_{1}}{V}\times 100 \end{array}$$

where V_1_ and V represent the volumes of the emulsified layer and the total liquid, respectively.

The emulsion was heated to 80°C for 30 min, cooled to 25°C, and centrifuged at 1,200 rpm for 5 min. The ES was calculated using Equation (12),


(12) $$\begin{array}{l} ES\left(mL/100mL\right)=\frac{V_{1}}{V}\times 100 \end{array}$$

where V_1_ and V are the volumes of the emulsified layer after and before, respectively.

The least gelation concentration (LGC) was measured based on a previous report with some modifications (34). The SDF powder was dissolved in distilled water to prepare the suspension with SDF concentrations of 2, 4, 6, 8, 10, and 12% (w/v), respectively. The suspensions (5 ml) were heated to 100°C and kept for 1 h, and then, they were placed in an ice bath for 1 h. The LGC was determined when the suspensions remained solidified even after inversion and shaking.

##### Thermal Analysis

The thermal properties were analyzed according to Einhorn-Stoll with minor modifications using a differential scanning calorimeter (35). Before determined, the instrument was calibrated with indium (Δ*H*_fusion_ = 23.86 J/g, melting point *T*_onset_ = 124.81°C). Compared with the reference of an empty pan, the dried SDF sample was heated from 30 to 150°C, at a rate of 5°C/min, with nitrogen input at 75 ml/min. All tests were repeated three times.

#### The Functional Properties of SDFs

##### The Adsorption Capacity of SDFs for Toxic Ions

The maximum binding capacity (BC_max_) and the minimum binding concentration (BC_min_) of the SDF for toxic ions were measured following the method previously reported with minor modifications (32). Before the adsorption test, the toxic ion solutions were prepared using Pb(NO_3_)_2_, CuSO_4_, and NaAsO_2_ to reach a concentration of 10 mmol/L for each. The adsorption test was carried out as follows: first, the SDF (1.0 g) was suspended in 100 ml of toxic ion solutions to obtain the mixture; to simulate the environments in the stomach and small intestine, the pH of the mixture was then adjusted to 2.0 and 7.0, respectively, and the mixture was shaken at 120 rpm at 37 °C for 3 h. Finally, 2 ml of the mixture and ethanol (8 ml) were centrifuged at 4,200 rpm for 25 min. The toxic ion concentrations in the supernatant were determined by inductively coupled plasma-atomic emission spectroscopy (Optima 2000 DV, Perkin-Elmer, Norwalk, CT, USA). The BC_max_ value for the toxic ions was calculated as follows:


(13) $$\begin{array}{l} BC_{\max}=\frac{\left(C_{0}-C_{S}\right)\times V}{W} \end{array}$$

where C_0_ is the concentration (μmol/L) of toxic ions added, C_s_ is the concentration (μmol/L) of toxic ions in the supernatant, V is the volume of the solution (mL), and W is the weight of SDF (g).

During the adsorption test, enough adsorption time leads to reaching the equilibrium concentration of the toxic ions (i.e., ions bound by SDF = ions released from SDF), which is BC_min_.

##### Measurement of *in vitro* Antioxidant Activities of SDFs

*DPPH and ABTS Radical Scavenging Assay*. 1,1-diphenyl-2-picrylhydrazyl radical scavenging capacity of the samples was evaluated based on the method of Zhu with slight modifications (36). DPPH was dissolved in methanol with a concentration of 6 ×10^−5^ mol/L, and 3 ml of DPPH solution was mixed with 100 μl of the SDF solution with some dilution. The mixture was kept for 20 min at 37°C in dark, and then, the decrease in absorbance due to adding the antioxidants (SDF) was measured under 515 nm. The experiment was repeated three times.

2,2′-azino-bis (3-ethylbenzothiazoline-6-sulfonic acid) was dissolved in deionized water to obtain a stock solution with 7 mmol/L. ABTS work solution was prepared, mixed with 2.45 mM potassium persulfate, and kept in the dark at 25°C for 12–16 h. ABTS^+^ solution was diluted in deionized water until an absorbance of 0.7 (±0.02) at 734 nm. Then, a 0.1 ml sample (appropriately diluted) and 3 ml ABTS·+ working solution were mixed for reaction in the dark for 30 min. The experiment was repeated three times (36). The percentage of the inhibition of ABTS^+^ was calculated using the following formula:

Free radical scavenging activity was calculated using the following formula:


(14) $$\begin{array}{l} scavengingactivity\left(\%\right)=\frac{\left(A_{c}-A_{E}\right)}{A_{c}}\times 100 \end{array}$$

where A_c_ and A_E_ are the absorbance of DPPH/ABTS without or with SDF, respectively.

*FRAP Assay*. The ferric ion reducing antioxidant power (FRAP) was examined as described by Xu (37). To obtain the working FRAP solution, 10 ml of acetate buffer (300 mmol/L, pH 3.6) and 1 ml of TPTZ (10 mmol/L) were mixed in 40 mmol/L of HCl solution, and 1 ml ferric chloride (20 mmol/L) was added. Then, a 1 μl sample solution and 300 μl of deionized water were mixed and diluted with 3 ml of freshly prepared FRAP solution, and the mixture was kept at 37°C for 30 min to measure the absorbance under 593 nm. The dose–response curves of FeSO_4_·7H_2_O were determined by the above method.

### Molecular Properties of SDFs

#### The Molecular Weight of SDFs

The molecular weight was measured based on the method published by Wang with slight modifications (31). In total, 60 mg of SDF was dissolved in 10 ml of NaNO_3_ (0.1 mol/L) and centrifuged for 10 min at 10,000 rpm. The supernatant was collected to measure the molecular weight distribution using a Waters™ 650E Advanced Protein Purification system (Waters Corporation, Milford, MA, USA). The sample size was set at 10 μl, and the speed was 0.5 ml/min. A standard curve was performed using bovine carbonic anhydrase (29,000 Da), horse heart cytochrome C (12,500 Da), aprotinin (6,500 Da), bacitracin (1,450 Da), gly-gly-tyr-arg (451 Da), and gly-gly-gly (189 Da). Based on the elution time of the calibration materials, the molecular weight could be calculated by a linear regression equation.

#### The Monosaccharide Composition of SDF

The monosaccharide composition of the SDF was measured based on the method reported by Wang with some adjustments (28). In total, 7 ml of sulfuric acid with a concentration of 6 mol/L was mixed with 0.15 g SDF and kept at 25°C for 1 h. After diluting the mixture, it was hydrolyzed at 100 °C for 1 h, and then, its pH was adjusted to 7.0 with NaOH (2 mol/L) and supplemented to 50 ml with deionized water. Afterward, the monosaccharide composition of the SDF was determined by HPAEC-PAD using a Dionex ICS-3000 chromatographic system (Dionex Co., Sunnyvale, USA). The column and mobile phase was Dionex CarboPac™ PA20 column (150 mm 3 mm, i.d., 5 μm) and 2 mmol/L of NaOH, and the elution flow rate was set at 0.4 ml/min.

### Surface Morphological Analysis

The scanning electron images (SEMs) of the SDFs with and without PEF treatment were gathered using a scanning electron microscope (JSM-6360LV, JEOL, Japan). Based on Peerajit's method (38), the SDFs were placed on a specimen holder with double-sided scotch tape and sputter-coated with gold (10 min, 2 mbar). Afterward, the samples were transferred to the scanning electron microscope with a setting of 15.0 kV accelerating voltage and observed under 1,000-fold magnification.

### Statistical Analysis

The software used was SPSS 20.0 (SPSS Inc., Chicago, USA). All results were expressed as mean ± S.D. Data were subjected to analysis of variance (ANOVA) and significant differences (*p* < 0.05) of means were analyzed with Duncan's multiple-range test. To assess the concordance between experimental and calculated values, the root-mean-squared deviation (RMSD) calculated using Equation (15) was adopted.


(15) $$\begin{array}{l} RMSD=\sqrt{\frac{1}{n}\overset{n}{\underset{i=1}{\sum }}{\left(\text{exp }erimental-calculated\right)}^{2}} \end{array}$$

## Results and Discussion

### Effect of PEF Treatment on Orange Peel

#### Effect of PEF Treatment on Cell Permeabilization in Orange Peel From Orange Peel

**Figure 2:**
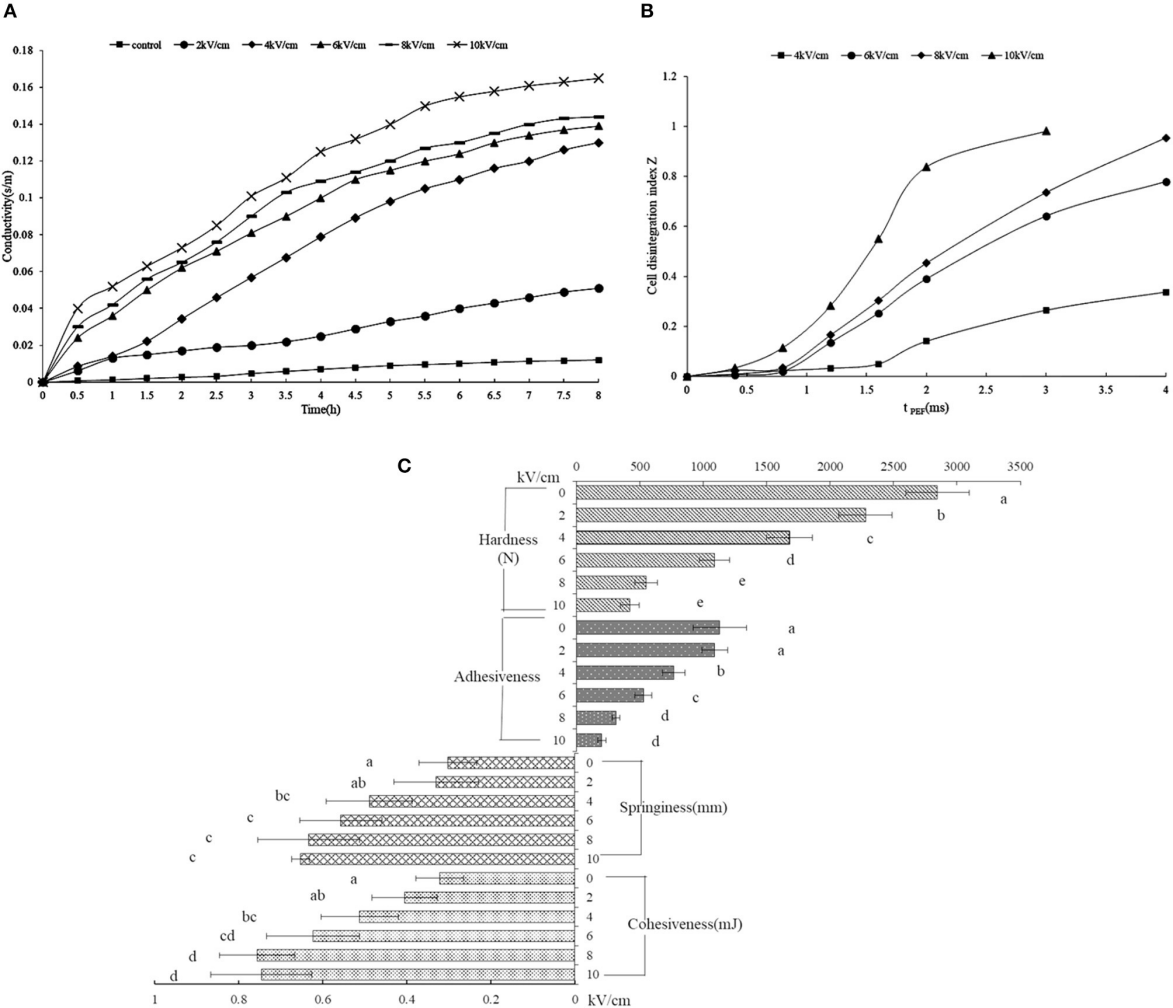
The rheological properties of SDF from the orange peel. **(A)** the storage and loss modulus for the SDF by PEF and control (G′ opened symbols, G^′′^ closed symbols); **(B)** viscosity for the SDF by PEF and control; **(C)** sheer stress for the SDF by PEF and control. PEF treatment parameters: electric field intensities 6.0 kV/cm, the number of pulses 30, the extraction time of 20 min, liquid-solid ratios of 15:1 and temperature of 45°C.

To estimate the membrane rupture, the released ions from the orange peel were adopted for examination (Figure 2A). The untreated peel showed a constant conductivity regardless of the length of time, indicating an intact membrane, while the consistently increasing conductivity of the PEF-treated peel solution indicated a destroyed membrane. The rapidly increasing conductivity depended on the electric field intensity above 2 kV/cm, which was clearly observed, indicating that a certain intensity leads to membrane disruption, sharply releasing the ionic material. Previous research also reported similar consistent results (19, 39). When the tissue possessed intact cell membranes, its electric resistance has a low conductivity value, while the penetrated cell membrane treated by PEF weakens the membrane resistance, finally leading to a change in cell impedance (40).

Figure 2B describes the degree of tissue disintegration through the electrical conductivity disintegration index *Z* values (20). The t_PEF_ (i.e., pulse width multiplied by the number of pulses) is dependent on the number of pulses. With the rise of PEF treatment time, the *Z* value showed a rapid increase, which started from a t_PEF_ of 1.6 ms (i.e., pulse number of 80) at 4 kV/cm, while the rapid increase began earlier at 0.8 ms (i.e., pulse number of 40) under a higher intensity (6–10 kV/ cm). The intact tissue shows a 0 value of Z, while 1 represents tissue that was ruptured thoroughly. As shown in Figure 2B, the intensity of 4 kV/ cm for some t_PEF_ could not destroy the cell; however, enhancing the intensity from 6 kV/cm resulted in tissue disintegration.

#### Effect of PEF Treatment on Orange Peel Textural Properties

Figure 2C shows the textural properties of the PEF-treated orange peel. The hardness values decreased obviously with the increasing field intensity. The hardness of the peel without PEF treatment was (2845.5 ± 176.8) N, which was 2.6, 5.2, and 6.8 times for 6, 8, and 10 kV/cm, respectively. A similar phenomenon was observed in the adhesiveness of the peel. In fact, apple, carrot, and potato tissues also showed stress-deformation properties after PEF treatment (41). In addition, both cell membranes and cell walls could be destroyed under high intensity (42). The cellulose contributes to the hardness of the cell wall, whose structure was destroyed by PEF, and the pectin in the intercellular layer, the main composition of SDF, was also degraded, thus, the hardness and adhesiveness decreased (43). With the rise of intensity, the springiness and cohesiveness of the peel showed an original increase and then a decrease. The springiness and cohesiveness of the PEF-treated peel were significantly higher with a higher intensity above 4 kV/cm than those without PEF treatment. The PEF destroyed the cellulose structure, causing a fracture, and the hemicellulose broke away from the cellulose, which could increase the hydrophilic swelling of the hemicellulose and decrease the limitation of the molecular chain space movement and rotation, leading to improved elasticity and cohesiveness (43).

### Effect of PEF Treatment on the Extraction Yield of SDFs

#### The Kinetic Model for PEF Assisted-Extraction of SDFs From the Orange Peel

**Figure 3:**
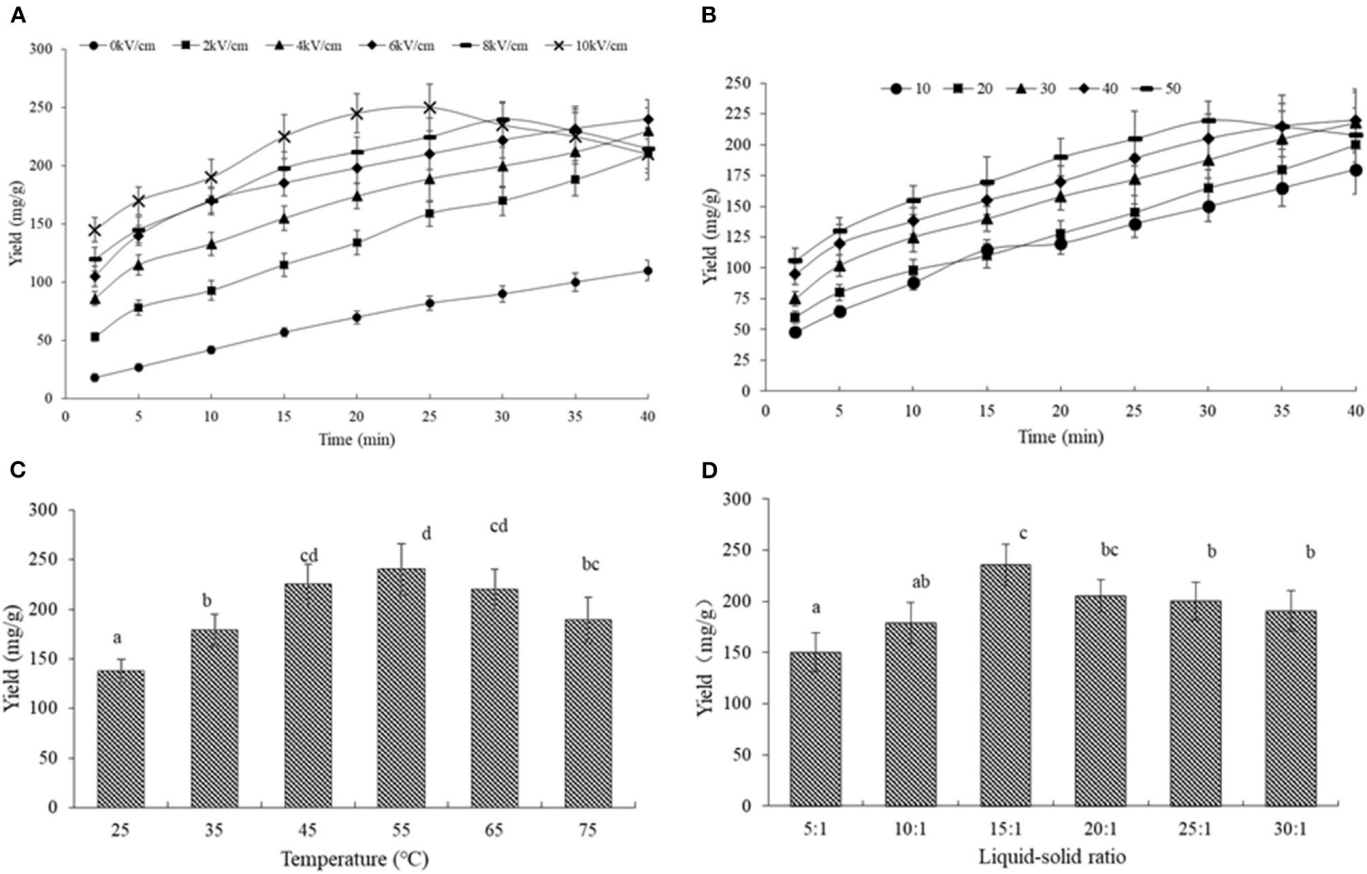
The effects of different parameters on the SDF yield from the orange peel. **(A)** SDF yield vs. time for all pulse electric field intensity with 30 number; **(B)** SDF yield vs. time for all pulse numbers at the constant of 6 kV/cm; **(C)** SDF yield for all temperatures of the constant of 6 kV/cm, 30 number and liquid-solid ratios of 15:1; **(D)** SDF yield for all liquid-solid ratios of the constant of 6 kV/cm, 30 number, and temperature of 45°C. Means with different letters are significantly different (*p* < 0.05).

Figures 3A,B describe the variation in the SDF yield with time, which was obtained with 10 to 50 pulses and electric field intensities from 0 to 10 kV/cm at a liquid-solid ratio of 15:1 and 45°C.

As shown in Figure 3A, the SDF treated with PEF showed a significantly higher yield than that without PEF treatment, indicating PEF's potential to improve the extraction efficiency of SDF, the accelerated extraction effects of PEF have been proven on a series of bioactive compounds such as naringin, polyphenols, pigments, and polysaccharides (11, 13, 16, 29). The main reason is the disintegration of the cell membrane under PEF treatment, a phenomenon that can be explained from the findings in Figure 2. Based on this penetrability, PEF assisted-extraction was also found to be very promising in pressed juices (such as apple juice, carrots, spinach, sugar beets, beans, artichokes, red cabbages, potatoes, onions, and grapes) (44).

From the kinetic curves in Figure 3A, the SDF rapidly increased during the whole extraction process under 0 and 2 kV/cm, the SDF obtained under 4 and 6 kV/cm sharply increased with time in the first 15 min, and then, it slightly increased, while, the peel treated under 8 and 10 kV/cm showed a strong increase in the SDF yield in the first 10 min, a slight increase from 15 to 30 min, and a decrease in the last 10 min. In a previous study, Rahaman approved that certain PEF intensities could improve extraction efficiency, while excessive intensities could not promote the extraction process (44). Moreover, the SDF yield treated with different pulse numbers showed a similar trend. The yield obtained with 50 started to decrease from 30 min (Figure 3B). Early studies reported that PEF treatment could accelerate some chemical reactions under room temperature and non-catalytic conditions including ethanol-acetic acid esterification and Feglycine complex synthesis (45). This was because PEF could promote mass transfer, which caused the variation in the polarization and structure of the molecules, leading to the occurrence of electrochemical and electrolytic reactions (46). On the other hand, the high amounts of energy delivered during electroporation by the electric pulses could lead to the deterioration and degradation of valuable compounds (47). Therefore, we speculated that the decrease in the SDF yield under higher intensity and pulses number would be related to the SDF decomposition and further chemical reactions between SDF or its decomposition product and other components in the orange tissues.

The values of the initial extraction rate (B_0_) and maximum content (Ce) for Peleg's model were calculated, as shown in Table 1. There was a very good agreement between the experimental data and those calculated by Peleg's model.

**Table 1:** The kinetic parameters of Peleg's model for SDF and comparison of experimental and calculated values.

**SDF yield**	**B_**0**_ (mg/g·min)**	**K_**1**_ (min·g/mg)**	**K_**2**_ (g/mg)**	**Ce (mg/g)**	***R* ^2^**	**RMSD**
**Pulse intensity**						
0 kV/cm	10.1523	0.0985	0.0098	102.0408	0.9431	0.0035
2 kV/cm	35.4610	0.0282	0.0057	175.4386	0.9134	0.0013
4kV/cm	68.9655	0.0145	0.0049	204.0816	0.9055	0.0007
6 kV/cm	91.7431	0.0109	0.0044	227.2727	0.9476	0.0004
8 kV/cm	116.2792	0.0086	0.0044	227.2727	0.9145	0.0005
10 kV/cm	185.1852	0.0054	0.0042	238.0952	0.8983	0.0004
**Pulse numbers**						
10	31.7460	0.0315	0.0064	156.2500	0.9161	0.0014
20	43.6681	0.0229	0.0062	161.2903	0.8724	0.0013
30	57.1429	0.0175	0.0052	192.3077	0.9154	0.0008
40	82.6446	0.0121	0.0050	200.0000	0.8729	0.0007
50	98.0392	0.0102	0.0048	208.3333	0.8993	0.0005

The Ce for the PEF-treated peel was 227.27 and 238.09 mg/g at the higher intensity above 4 kV/cm, which was 2.2 and 2.3 times higher than that without PEF, respectively. Moreover, the Ce increased rapidly from 10 to 30 pulses, and then, it slightly increased. An increase over 8.0 kV/cm or over 40 pulses had an adverse effect on SDF extraction. Therefore, the intensity of 6 kV/cm and a pulse number of 30 was selected in the subsequent experiments, based on the experimental results as well as the *R*^2^ and RMSD values.

#### Effect of Temperature on the Extraction Yield of SDF

As shown in Figure 3C, the SDF yield increased from 25 to 55°C with the highest yield of 230.1 mg/g, while it decreased to 180 mg/g at 75°C. It is well-known that a higher temperature promotes solubility, resulting in a high yield. Meanwhile, an elevated temperature could lower the solution's viscosity leading to a minimization of the mass-transfer resistance (48). Under PEF, a high temperature could improve the solvent's diffusivity, strengthen the sample wetting, and promote matrix penetration (49). Nonetheless, the relationship between the degree of esterification (DE) and temperature appeared to be negatively correlated at higher temperatures, it was speculated that the decreasing SDF yield was due to the de-esterification of the methoxyl group in the pectin chain leading to the molecule degradation.

#### Effect of Liquid-Solid Ratio on the Extraction Yield of the SDF

Figure 3D shows that the maximum SDF yield of 238 mg/g was obtained with a liquid-solid ratio of 15:1. The yield increased initially, but it gradually declined at a higher liquid-solid ratio. When the liquid-solid ratio was increased, the dissolving capacity was accordingly increased, once the liquid-solid ratio increased to 15:1, the yield decreased with the reduced ability to separate SDF from the solution (50) due to lower protection for the dissolved SDF and the higher degradation (51).

### The Processing Properties of SDFs

#### The Rheological Properties of SDFs

**Figure 4:**
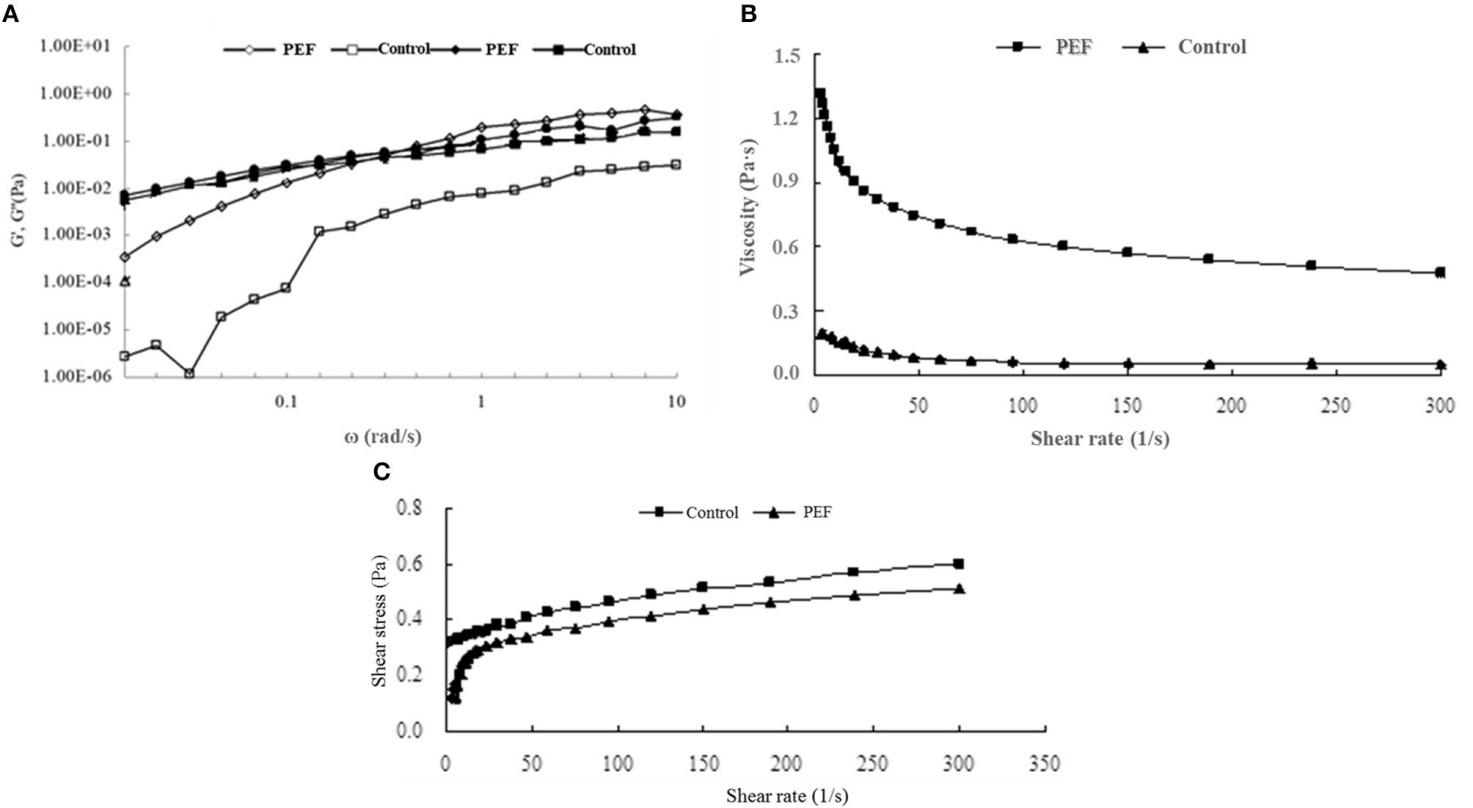
Effects of PEF and control SDF from orange peel on antioxidant activity. **(A)** DPPH radical scavenging capacities; **(B)** ABTS radical scavenging capacities; **(C)** ferric ion reducing capacity. Mean values with different letters are significantly different (*p* < 0.05). PEF treatment parameters: electric field intensities 6.0 kV/cm, the number of pulses 30, the extraction time of 20min, liquid-solid ratios of 15:1 and temperature of 45°C. The results were expressed as mean ± standard deviation (*n* = 3), the different letters indicated that the difference was significant (*P* < 0.05) and the same letter was expressed as insignificant difference.

Figure 4A illustrates the viscoelastic behavior of the SDF. The increasing value of G' with the rise in the frequency was almost frequency-dependent. Compared with the value of G' at a low frequency, G” showed a higher value, indicating a liquid-like behavior. In a previous report, citrus fibers also showed similar characteristics to viscoelastic fluids (52). With the rise of frequency, G' and G” were crossed, then, the G' value exceeded G” indicating a gel-like behavior, contributing to the overall dough elasticity and strength (53).

Viscosity is a main physicochemical property of SDF, including gums, pectins, psyllium, and β-glucans (54), which is related to the adsorption capacity. Figure 4B shows the viscosity of the SDF of orange peel. It was observed that the SDF obtained with PEF had a higher viscosity than the control, and during a lowshear rate range below 100 s^−1^, the viscosity of PEF-SDF decreases obviously as the shear rate increases, which showed a shear-thinning phenomenon and seemed to be a pseudoplastic fluid. When static or at a low flow rate, they hook and tangle with each other leading to higher viscosity. The pseudoplastic behavior is due to the disentanglement of the polymer network and the partial chain orientation in the shear flow direction (55). After a further increase in shear rate, the interactions between the molecules may be diminished owing to a micro-structural anisotrophy arising from shear deformation, thus which lowers the viscosity. The same shear-thinning phenomena were observed from orange pectin (56, 57). Compared with the viscoelastic behavior of control-PEF, a higher degree of shear-thinning of PEF-SDF was observed. It is well-confirmed that pectin plays the main role of SDF (58, 59). Pasandide found that the pectin solution behavior was different in various concentrations. When in low concentrations of pectin solution, the flow behavior was Newtonian, and the viscosity remained nearly constant with the increasing shear rate. With pectin concentration increasing, the flow behavior changed from Newtonian to pseudoplastic and the viscosity was decreased with an increase in shear rate (55). The finding was accordant with our results. Therefore, it was speculated that the control SDF seem contained less pectin, the main component of the SDF, while, PEF treatment could improve the yield of the SDF including the pectin.

Based on the high viscoelastic behavior of PEF-SDF, it was reported to decrease the cholesterol level (60), which agreed with our finding of the higher efficiency of the SDF with PEF treatment in attenuating cholesterol concentration than the control (Table 2).

**Table 2:** Effects of PEF^A^ on physicochemical properties of orange peel SDF.

**Sample**	**WS**	**WHC**	**OHC**	**SC**	**EA**	**ES**	**LGC**
	**(%)**	**(g/g)**	**(g/g)**	**(mL/g)**	**(mL/100 mL)**	**(mL/100 mL)**	**(%)**
Untreated	79.35 ± 1.29^a^	3.49 ± 0.32^a^	2.04 ± 0.28^a^	5.07 ± 0.48^a^	61.58 ± 2.48^a^	42.58 ± 1.77^a^	11.58 ± 0.39^a^
PEF-SDF	90.12 ± 1.11^b^	5.67 ± 0.67^b^	3.89 ± 0.41^b^	6.96 ± 0.88^b^	79.69 ± 2.36^b^	70.36 ± 2.13^b^	8.18 ± 0.28^b^

*The values represent means of triplicates ± standard deviation. Values in the same column with different letters are significantly different (p <0.05). WS, water solubility; WHC, water-holding capacity; OHC, oil-holding capacity; SC, swelling capacity; EA, emulsifying activity; ES, emulsion stability; LGC, least gelation concentration.*

A *PEF treatment parameters: electric field intensities 6.0 kV/cm, the number of pulses 30, the Liquid-solid ratio of 15:1, extraction time of 20 min, and temperature of 45°C*.

In Figure 4C, the shear stress of the control showed an almost linear dependence on the shear rate, indicating approximately Newtonian fluid, and the control had higher yield stress perhaps due to its longer molecular structure and larger particle size. Lower yield stress of the PEF-SDF proved that it showed a smaller particle size (52). Therefore, it is speculated that PEF treatment was beneficial to the smaller molecules' structure due to its ability to promote the ability of cell fracture.

#### Solubility, Water-and Oil-Holding Capacities, and Swelling Capacity

The water solubility, water-holding capacity, and swelling capacity of the SDF significantly affect the rheological properties of the functional foods which contribute to the mouth-feel experience (61).

The effect of PEF pretreatment on the WS, WHC, OHC, and SC of the SDF is shown in Table 2. In general, the polysaccharide constituents of the dietary fibers were strongly hydrophilic. After being treated with PEF, the WS of the SDF was increased (79.35 ± 1.29% vs. 90.12 ± 1.11%), and PEF treatment also significantly increased the WHC from 3.49 ± 0.32 g/g db to 5.67 ± 0.67 g/g db (*p* < 0.05). In addition, the SC of the SDF from orange peel treated by PEF was increased from 5.07 ± 0.48 mL/g db to 6.96 ± 0.88 mL/g db. These water-related properties were partly dependent on the individual components of the dietary fiber and its physical structure (such as porosity and crystallinity) (62). The larger value of the WS, WHC, and SC shown by the SDF treated with PEF is speculated to be associated with a rise in the proportion of short-chain dietary fiber and its surface area.

The oil-holding capacity of the SDF referring to the retaining of oil is important for its application in functional foods, including proceeding properties and nutrition value (63). As shown in Table 2, treating with PEF increased the OHC 1.91-fold compared with the control SDF. It was concluded that the SDF from orange peel treated by PEF might be a better selection for functional foods.

#### Emulsifying Activity, Emulsion Stability, and Least Gelation Concentration

The emulsifying properties including the emulsifying activity (EA) and emulsion stability have important functions, referring to facilitating solubilization or dispersion and resistance to rupture of the emulsion (64).

The SDF showed an EA property *via* its interfacial absorption with a high-tensile strength, which could promote the formation of condensed films. The formed films around fat droplets could resist fat droplet coalescence due to the hydrophilic barrier in the interface between the oil and water phases (65).

As shown in Table 2, the EA values of the SDF were 61.58 ± 2.48 mL/100 ml and 79.69 ± 2.36 mL/100 ml obtained without or with PEF, respectively. Similarly, the SDF obtained with PEF showed a larger value than that without PEF (42.58 ± 1.77 mL/100 ml vs. 70.36 ± 2.13 mL/100 ml). This indicated that SDF-PEF might be a better emulsifying agent.

The gelation capacity, assessed by the LGC, is the main property for the acceptability of related food. In general, a lower LGC value represents a better gelation property. The LGCs of the SDF from untreated and treated by PEF were 11.58 ± 0.39% (w/v) and 8.18 ± 0.28% (w/v), respectively, which indicated that the SDF obtained with PEF showed an excellent gelation property.

#### Thermal Analysis

**Figure 5:**
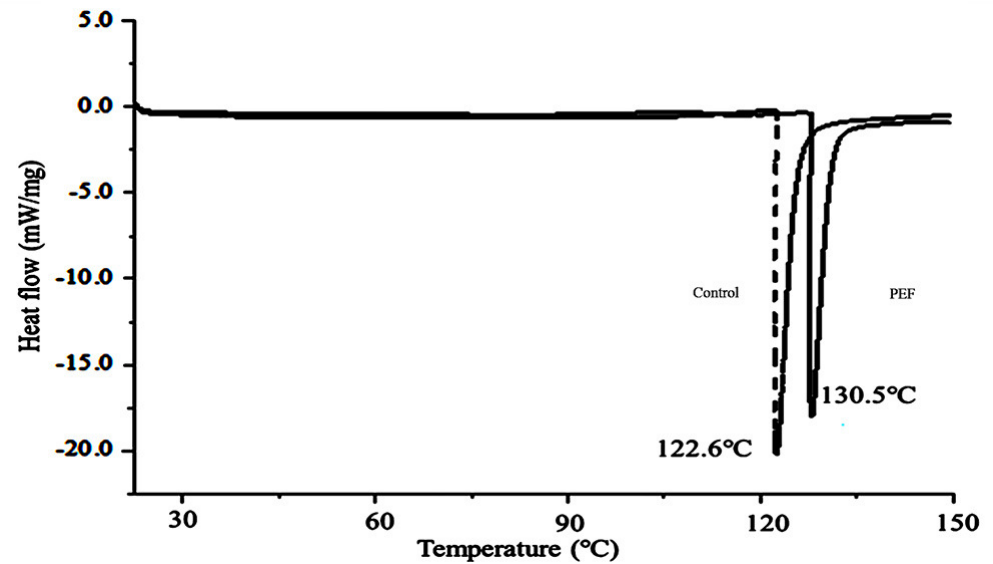
The DSC analytical curve of the SDF from orange peel (solid line: treated by PEF, dotted line: untreated). PEF treatment parameters: electric field intensities 6.0 kV/cm, the number of pulses 30, the extraction time of 20 min, liquid-solid ratios of 15:1 and temperature of 45°C.

The thermodynamic properties of the SDF were estimated by DSC. As shown in Figure 5, the SDF without or with PEF treatment had endotherms curves, whose peak temperatures were 122.6 and 130.5°C, respectively. A similar phenomenon was reported for soybean residue SDF (66). The previous research found that short-chain SDF had strong hydrogen bonds that required more energy to decompose its crystalline structure (67). Therefore, this could explain the higher content of short-chain SDF-PEF promoting an increase in the peak temperature. Consistently, no change in the exothermic and endothermic processes was observed between the SDFs with different treatments until a temperature above 150°C, indicating the excellent thermal stability of SDF from orange peel.

### The Functional Properties of the SDF

#### The Binding Capacity of Three Toxic Ions to the SDF

The maximum binding capacity (BC_max_) and the minimum binding concentration (BC_min_) were adopted to evaluate the adsorption capacity of the SDF for the toxic ions.

From Table 3, the SDFs showed a larger vale of BC_max_ and a smaller value of BC_min_ at pH 7.0 rather than pH 2.0. Under pH 7.0, the BC_max_ values were two to three times higher than those at pH 2.0, and the BC_mix_ values were sharply decreased for the two types of SDFs. It is well-known that a pH of 7.0 is similar to the intestinal environment, which indicates that SDFs had a high affinity for the toxic cations in the intestinal tract. In fact, mineral absorption does not occur in the stomach, while foods and toxic materials remain much longer in the small intestine, which is the main organ to absorb minerals. Compared with the control SDF, SDF-PEF exhibited a larger and smaller value of BC_max_ and BC_min_, respectively, which shows that SDF-PEF provides more protection from the heavy metals that can cause damage to the body. The mechanical sorption of the SDF depends on the degree of porosity (68), and it is speculated that the better sorption capacity of SDF-PEF was due to higher porosity to trap the toxic cations. Interestingly, the morphology of SDF-PEF supported this speculation, as shown in **Figure 8** in this study.

**Table 3:** Effects of PEF^A^ on the values of the maximum binding capacity (BC_max_) and the minimum binding concentration (BC_min_) of the orange peel SDF for Pb, As, and Cu at pH 2.0 and pH 7.0.

**Sample**	**pH**	**BC**_**max**_ **(μmol/g)**			**BC**_**min**_ **(μmol/L)**		
		**Pb**	**As**	**Cu**	**Pb**	**As**	**Cu**
Untreated	2.0	68.6 ± 2.1^a^	50.7 ± 1.2^a^	42.8 ± 1.3^a^	225.2 ± 6.1^a^	296.8 ± 6.3^a^	268.4 ± 8.2^a^
PEF	2.0	182.4 ± 4.2^b^	155.2 ± 3.4^b^	102.4 ± 3.7^b^	153.7 ± 3.2^b^	159.8 ± 5.4^b^	153.7 ± 6.1^b^
Untreated	7.0	208.9 ± 3.8^a^	182.5 ± 4.1^a^	86.3 ± 3.2^a^	105.5 ± 4.3^a^	113.7 ± 4.7^a^	127.3 ± 4.7^a^
PEF	7.0	359.2 ± 5.8^b^	315.7 ± 4.7^b^	177.5 ± 4.9^b^	55.8 ± 2.1^b^	69.8 ± 3.5^b^	88.9 ± 2.8^b^

*The values represent means of triplicates ± standard deviation. Values in the same column with different letters are significantly different (p <0.05).*

A *PEF treatment parameters: 6.0 kV/cm, the number of pulses 30, the liquid-solid ratio of 15:1, extraction time of 20 min, and temperature of 45°C*.

#### *In vitro* Antioxidant Activities of SDF

**Figure 6:**
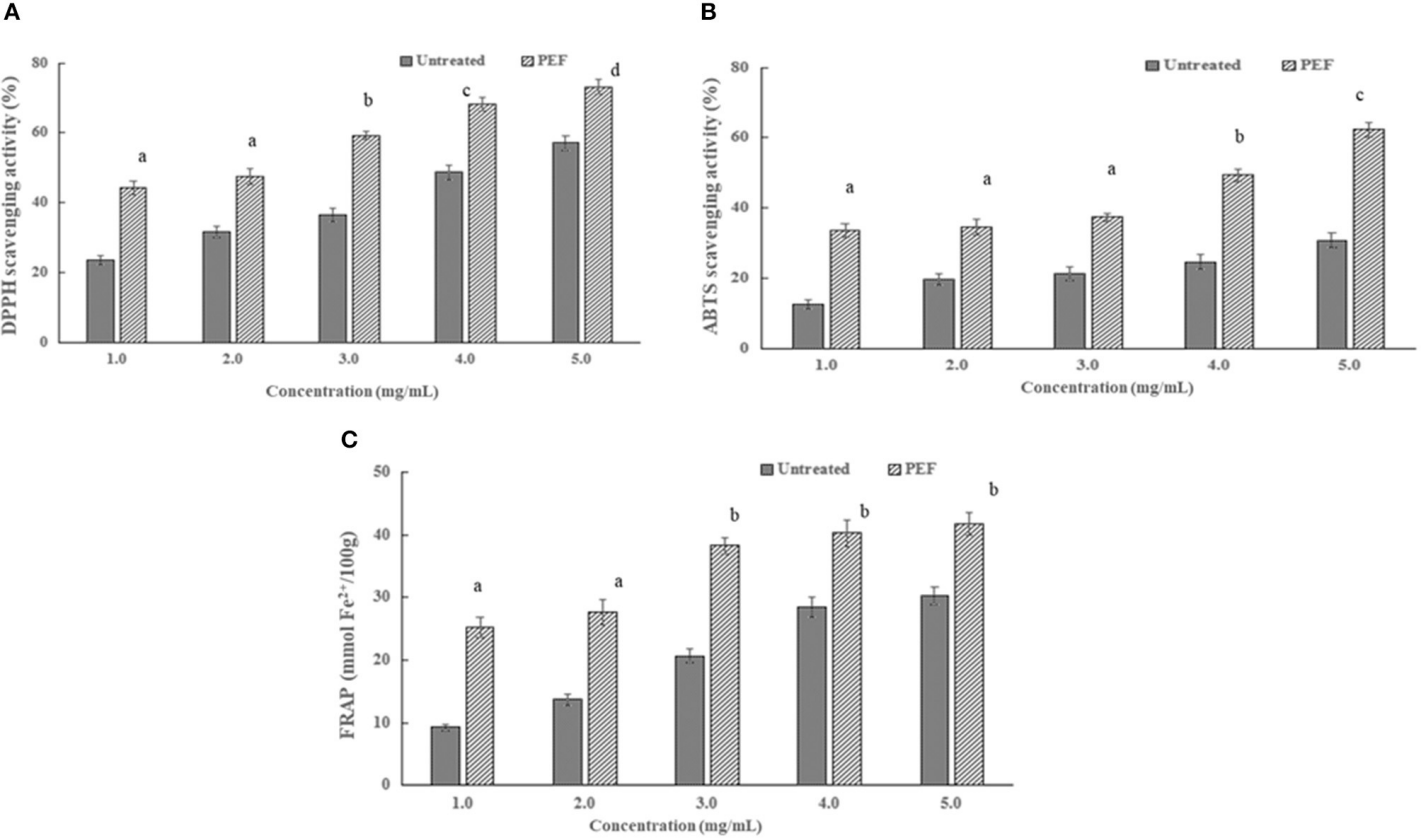
Effect of pulsed electric field treatment on the micro- and macrochange of the orange peel. **(A)** the ion release from orange peel; **(B)** orange peel cell membrane disintegration index [the total PEF treatment time (t_PEF_) was calculated by the equation t_PEF_ = (pulse number, n) × (pulse width, s)]; **(C)** the texture properties of the orange peel. The results were expressed as mean ± standard deviation (*n* = 3), the different letters indicated that the difference was significant (*P* < 0.05) and the same letter was expressed as insignificant difference.

As seen in Figure 6, there are similar dose-dependent trends of the radical scavenging activity of the SDFs at a concentration above 2 mg/ml. In addition, the scavenging activity of the DPPH and ABTS radical of SDF-PEF was stronger than the control SDF, accordingly, the stronger metal-reducing capacities belonged to the SDF-PEF. The highest scavenging activity of 73.2% for DPPH radical and 62.4% for ABTS radical was exhibited at the SDF-PEF concentration of 5.0 mg/ml, which was higher than those of the control SDF. It was generally concluded that PEF-SDF possessed a good extent reducing power and radical scavenging capacity, and might limit the occurrence of free radical damage in the human body.

### Molecular Characterization of the SDF

#### The Molecular Weight Distribution of the SDF

**Figure 7:**
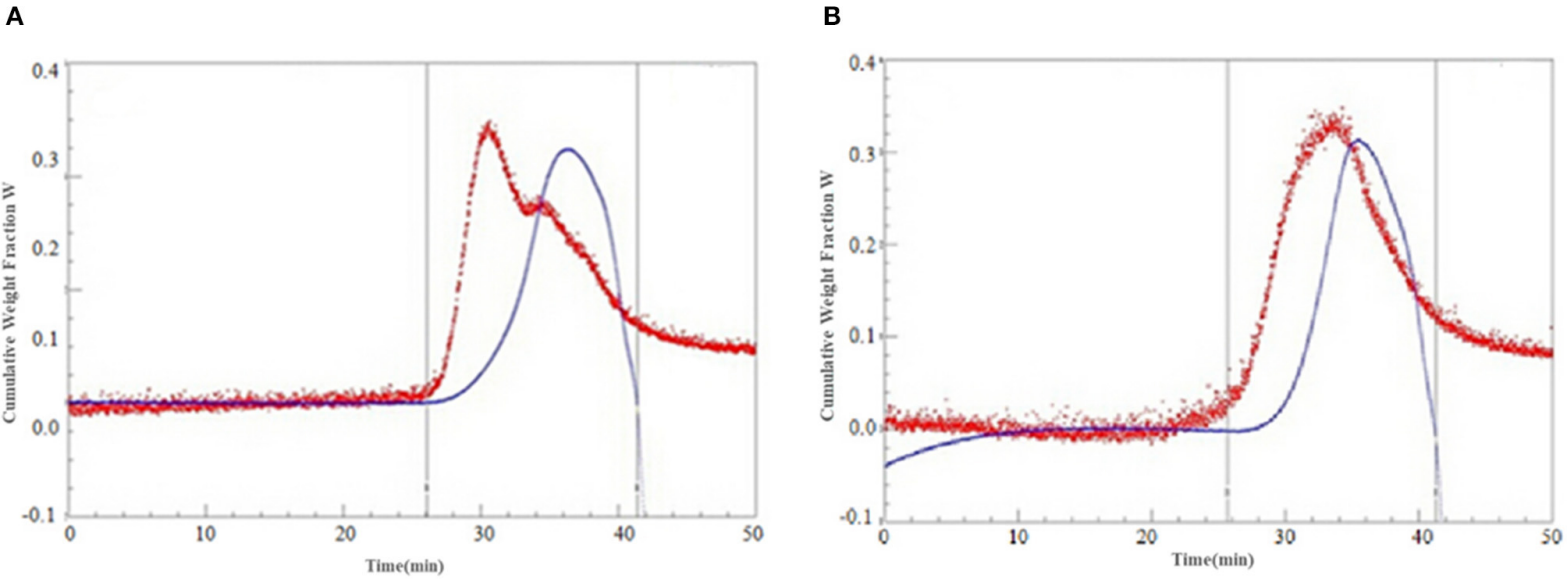
The cumulative weight fraction of the SDF from orange peel [**(A)** Control-SDF, **(B)** PEF-SDF]. PEF treatment parameters: electric field intensities 6.0 kV/cm, the number of pulses 30, the extraction time of 20min, liquid-solid ratios of 15:1 and temperature of 45°C.

The molecular weights of the SDF are shown in Table 4 and Figure 7. Compared with the control SDF with a weight-average molecular weight of 512 kDa, the PEF pretreatment obviously decreased the molecular size distributions with a weight-average molecular weight of 189 kDa (*p* < 0.05), which implied that PEF treatment can severely degrade the IDF, such as cellulose, part of hemicellulose and lignin. The number-average molecular weight (Mn) exhibited corresponding changes with the Mw. In addition, the smaller value of polydispersity belonged to the SDF-PEF, indicating narrow polydispersity.

**Table 4:** Effects of PEF^A^ on the molecular weight of orange peel SDFs.

**Sample**	**Weight-average molecular weight Mw^**a**^ (kDa)**	**Number-average molecular weight Mn^**b**^ (kDa)**	**Polydispersity Pd^**c**^ (Mw/Mn)**
SDF from untreated orange peel	512	178	2.88
SDF from PEF-treated orange peel	189	102	1.85

*Values are given as means of independent experiments.*

A *PEF treatment parameters: electric field intensities 6.0 kV/cm, the number of pulses 30, the Liquid-solid ratio of 15:1, extraction time of 20 min and temperature of 45°C.*

*The results were expressed as mean ± standard deviation (n = 3), the different letters indicated that the difference was significant (P <0.05), and the same letter was expressed as insignificant difference*.

#### Monosaccharide Constituents of the SDF

Table 5 shows the monosaccharide composition of the SDF. The main monosaccharides for the SDF were arabinose and glucose, while the contents of arabinose and glucose in the SDF with PEF treatment were higher than that in the control SDF, which suggests that the different extraction methods did not change the type of monosaccharide but could change the monosaccharide content. It was speculated that the PEF treatment might result in changes in the molecules' structure reflecting the degree of branching and linearity of the main chains (31). Compared with the control SDF, the SDF with the PEF treatment contained a large number of neutral sugars and the RG-I region (2 Rha+ Ara+ Gal) and a smaller proportion of the HG region (GalA-Rha), indicating a lower degree of linearity (69), the branching of the RG-I region, and a larger degree of branching, which exhibited more bondage chains and more intermolecular forces including a hydrogen bond, hydrophobic interaction, etc., improving the viscosity and viscoelasticity (4, 70).

**Table 5:** Effects of PEF^A^ on monosaccharide constituents of orange peel SDFs.

	**SDFs**	
**Monosaccharide (mg/g db^**B**^)**	**PEF-SDF**	**Control-SDF**
Rhamnose (Rha)	14.47 ± 1.24	14.34 ± 1.24
Arabinose (Ara)	78.32 ± 5.21	74.72 ± 6.12
Galactose (Gal)	16.13 ± 1.34	16.8 ± 1.22
Glucose (Glu)	9.17 ± 0.64	11.04 ± 1.04
Xylose (Xyl)	4.32 ± 0.54	7.68 ± 3.67
Mannose (Man)	7.52 ± 0.54	9.06 ± 0.74
Fructose (Fru)	13.48 ± 1.56	15.40 ± 1.39
Galacturonic acid	39.48 ± 2.54	38.23 ± 2.87
GalA/Rha	2.72 ± 0.15	2.67 ± 2.10
GalA/(Fuc+ Rha+ Ara+ Gal+Xyl)	0.13 ± 0.04	0.15 ± 0.07
HG= GalA- Rha	25.01 ± 3.04^a^	34.96 ± 2.97^b^
RG-I=2 Rha+ Ara+ Gal	113.49 ± 13.10^c^	95.2 ± 12.12^d^

*The values represent means of triplicates ± standard deviation.*

A *PEF treatment parameters: electric field intensities 6.0 kV/cm, the number of pulses 30, the Liquid-solid ratio of 15:1, extraction time of 20 min and temperature of 45°C.*

B *db, dried base.*

*The different letters indicated that the difference was significant (P <0.05) and the same letter was expressed as insignificant difference*.

### Scanning Electron Morphology

**Figure 8:**
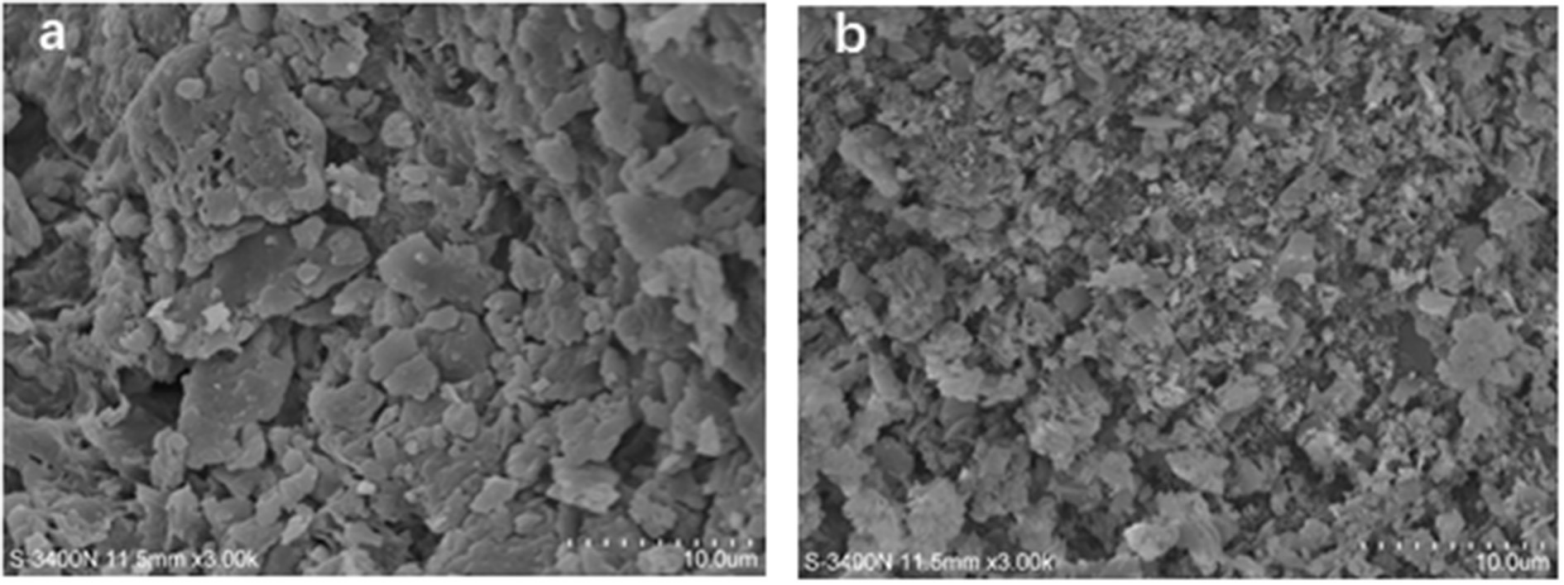
Scanning electron microcopy (SEM) images of the SDF from orange peel [**(a)** control, **(b)** treated by PEF at electric field intensities 6.0 kV/cm and the number of pulses 30). PEF treatment parameters: electric field intensities 6.0 kV/cm, the number of pulses 30, the extraction time of 20 min, liquid-solid ratios of 15:1 and temperature of 45°C.

The surfaces of the untreated-SDF and PEF-SDF were analyzed using SEM, and the results are shown in Figure 8. The surface of the untreated SDF appeared as a large slice out of the structure (Figure 8a). The surface of the PEF-SDF with a small slice was concave and exhibited a rough structure (Figure 8b). It is speculated that PEF treatment damaged the groove structure, causing more flakes, holes, and cracks, thus increasing the exposure of the internal structure of the SDF. Therefore, the spatial structure and the regiochemistry properties were likely changed by the PEF treatment, which could be accounted for the excellent physiological properties of dietary fiber including adsorption or binding of some molecules (71).

### The Mechanism of PEF Assisted-Extraction of SDF

**Figure 9:**
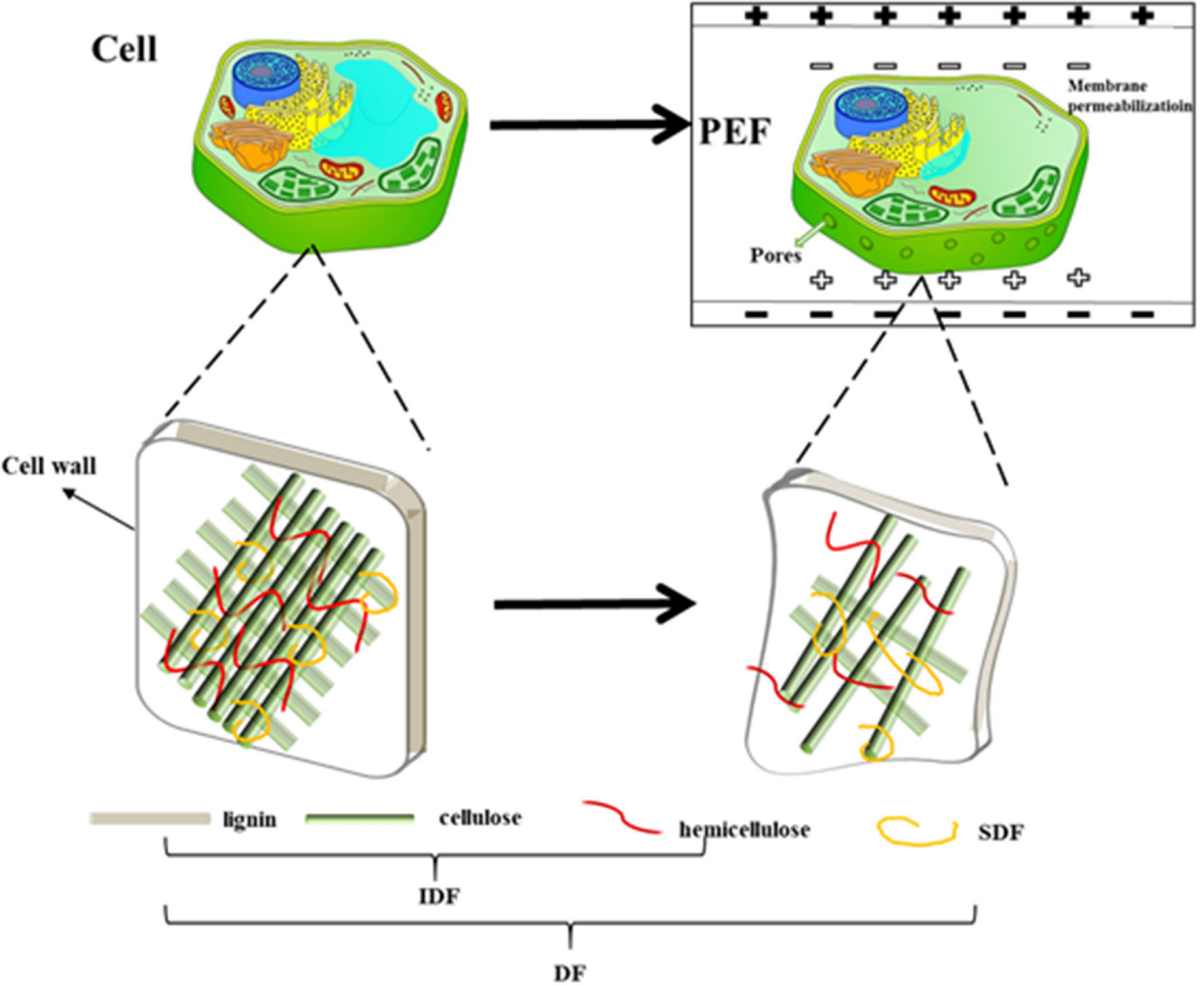
The mechanism of PEF assisted-extraction of the SDF from orange peel.

Generally, the mechanism of PEF assisted-extraction of SDF from orange peel involved two aspects (Figure 9). On the one hand, the ions in the cytoplasm moved under PEF to generate a potential difference on both sides of the cell membrane. When the electric field intensity exceeded the threshold value, the cell membrane formed irreversible electroporation (72), which accelerated the release of SDF from the orange peel. On the other hand, the PEF treatment resulted in a weaker intermolecular and intermolecular hydrogen bond of the cellulose in the cell wall of the peel, it also significantly reduced its crystallinity and rigidity, which made it impossible to support and reinforce the structure. Furthermore, the cellulose changed from a cluster fiber aggregation structure to dispersed a single cellulose chain, and many gaps on its surface, which cause the pectin and hemicellulose to degrade and dissolve. As such, it easily resulted in cell wall rupture and cytoskeleton destruction. In addition, PEF treatment decreased the molecular weight and viscosity of the pectin, leading to a weakened adhesion force between the cell walls, which resulted in cell wall rupture due to natural turgor pressure and tension. In summary, PEF treatment resulted in the perforation of the cell membrane, the collapse of the cell wall, and the release of SDF such as pectin.

## Conclusions

The pulsed electric field pretreatment for orange peel was an efficient method for improving the SDF yield and physicochemical properties under optimal conditions (electric field intensity 6.0 kV/cm and 20 pulses). The main weight-average molecular weight (Mw) of SDF from PEF-treated orange peel (189 kDa) was significantly (*p* < 0.05) lower than that of the untreated SDF (512 kDa). The SDF-PEF revealed a tough, irregular surface with a porous structure and high thermal stability. Therefore, PEF technology is a preferable choice for the production of active SDF from orange peel.

## Data Availability Statement

The raw data supporting the conclusions of this article will be made available by the authors upon request.

## Author Contributions

RF and LW contributed to the design and investigation of the study, performed the statistical analysis, and wrote the first draft of the manuscript. LW helped in funding acquisition. JF and WS performed the statistical analysis and organized the figures. HD and RF contributed to supervision, visualization, and writing—review and editing. All authors contributed to manuscript revision, read, and approved the submitted version.

## Funding

This work was supported by key science and technology projects in Hebei province of China (No. 19227516D) and China Postdoctoral Science Foundation - Chian (2019BH029).

## Conflict of Interest

WS was employed by Beijing institute of nutritional resources Co., Ltd. The remaining authors declare that the research was conducted in the absence of any commercial or financial relationships that could be construed as a potential conflict of interest. The authors declare that this study received funding from key Science and Technology Projects of Hebei Province of China (No. 19227516D) and China Postdoctoral Science Foundation - China (2019BH029). The funder was not involved in the study design, collection, analysis, interpretation of data, the writing of this article, and the decision to submit it for publication.

## Publisher's Note

All claims expressed in this article are solely those of the authors and do not necessarily represent those of their affiliated organizations, or those of the publisher, the editors and the reviewers. Any product that may be evaluated in this article, or claim that may be made by its manufacturer, is not guaranteed or endorsed by the publisher.
